# Human Metapneumovirus: A Largely Unrecognized Threat to Human Health

**DOI:** 10.3390/pathogens9020109

**Published:** 2020-02-13

**Authors:** Charles J. Russell, Rhiannon R. Penkert, Sonnie Kim, Julia L. Hurwitz

**Affiliations:** 1 Department of Infectious Diseases, St. Jude Children’s Research Hospital, Memphis, TN 38105, USA; charles.russell@stjude.org (C.J.R.); rhiannon.penkert@stjude.org (R.R.P.); 2 Department of Microbiology, Immunology and Biochemistry, University of Tennessee Health Science Center, Memphis, TN 38163, USA; 3 National Institute of Allergy and Infectious Diseases, National Institutes of Health, Rockville, MD 20852, USA; sonnie.kim@nih.gov

**Keywords:** respiratory virus infection, Sendai virus, vaccine development

## Abstract

Human metapneumovirus (HMPV) infects most children by five years of age. The virus can cause both upper and lower respiratory tract disease and can be life threatening. High-risk populations include young children who are exposed to virus for the first time and the elderly. Currently, there is no standard treatment nor licensed vaccine for HMPV, although several attractive vaccine candidates have been developed for pre-clinical studies. A raised awareness of the impact of HMPV on public health is needed to drive research, complete vaccine development, and thereby prevent significant virus-associated morbidities and mortalities worldwide.

## 1. The Virus

Human metapneumovirus (HMPV) is a negative-strand RNA virus that replicates in the cytoplasm [1]. HMPV and avian metapneumovirus (AMPV), the closest known relative of HMPV, are members of the genus *Metapneumovirus* and the family *Pneumoviridae* [2]. HMPV shares many features with respiratory syncytial virus (RSV) of the genus *Orthopneumovirus* and family *Pneumoviridae* [3]. The HMPV genome is approximately 13,000 nucleotides in length. HMPV virions contain a ribonucleocapsid core consisting of viral RNA (vRNA), nucleocapsid (N) protein, polymerase (L) protein, phosphoprotein (P), and M2-1 protein [4]. The matrix (M) protein lines the inner face of the virion envelope. Envelope spikes projecting from the virus include a small hydrophobic (SH) protein and the attachment (G) and fusion (F) glycoproteins. An M2-2 protein regulates RNA synthesis and supports virus growth in a hamster model [5]. HMPV exists as filaments and spherical particles with diameters that range from 150 to 600 nm [1]. It appears morphologically similar to RSV and to members of the family *Paramyxoviridae*. In fact, HMPV and RSV were previously classified as members of the *Paramyxoviridae* family; listings were recently changed by the International Committee on Taxonomy of Viruses (ICTV) [6]. 

HMPV envelope glycoproteins include the G and F proteins. The open reading frame (ORF) of G encodes a 229–236 residue glycoprotein that is unrelated in nucleotide or amino acid sequence to other pneumovirus or paramyxovirus G proteins [4]. The G protein binds heparan sulfate and other glycosaminoglycans found on airway epithelial cells in nasal and lung tissues to initiate viral entry [7,8]. 

The ORF of F encodes a 539 residue glycoprotein that shares 33%–38% amino acid identity with other pneumovirus F proteins and 10%–18% identity with paramyxovirus F proteins [4]. As is the case for other pneumoviruses and paramyxoviruses, the F protein is synthesized as an F0 precursor protein that is subsequently cleaved into fusion-capable F1 and F2 subunits that form a large, trimeric, mushroom-like extracellular head in addition to C-terminal transmembrane (TM) and cytoplasmic tail regions. High-resolution structures have been obtained for the HMPV F protein ectodomain in both prefusion and postfusion forms [9,10]. These structures share many structural features common to prefusion and postfusion forms of the F proteins from RSV and paramyxoviruses [11,12,13,14]. After receptor binding, the F protein undergoes a dramatic change in structure that causes membrane fusion to allow virus entry. Most strains of HMPV catalyze membrane fusion at neutral pH [15] while others are triggered by low pH in endosomes [16,17]. In addition to membrane fusion, the F protein can also facilitate receptor binding through an Arg-Gly-Asp (RGD) motif that binds integrins [18,19]. 

## 2. HMPV Discovery, Diagnoses, and Prevalence

HMPV was discovered in 2001 after isolation from dozens of young children in the Netherlands [1]. Soon after its initial identification, HMPV was found globally as an etiological agent of respiratory infections in young children and elderly adults [20,21,22,23]. In temperate climates, HMPV epidemics tend to occur in winter and spring while in tropical climates, they are more sporadic [24,25,26,27,28].

Polymerase chain reaction (PCR) assays and immunofluorescence assays are often used to diagnose HMPV [29,30]. These assays can score the viral nucleic acids and proteins of an acute infection and they may also score residual viral components after replication-competent virus has been cleared. Serum anti-viral antibodies serve as another marker of HMPV exposure, because the B cell response to HMPV is rapid and long-lasting [29,30]. Serological findings suggest HMPV has been circulating globally in humans for decades or more before its first discovery, and that most children are first infected with HMPV before the age of five [31,32,33], with nearly 100% of teenagers and adults being seropositive [29,34]. The late discovery of HMPV was likely due to the virus’s slow replication, requirement for trypsin, and minimal cytopathic effects (CPE) in many tissue culture cell lines. HMPV disease was often attributed to another virus such as RSV or influenza. Tertiary monkey kidney epithelial cells were eventually found to support robust HMPV amplification and CPE [1]. 

## 3. The Disease and Risk Factors

Following HMPV exposure, an innate cell, B cell, and T cell immune response usually develops within days and assists rapid virus clearance [29,30]. However, HMPV can cause both upper respiratory tract (URT) and lower respiratory tract (LRT) disease, in some cases exacerbated by immune function [35,36]. LRT disease can be severe, particularly when an individual is exposed to virus for the first time. Outcomes can include bronchiolitis and pneumonia and can result in death [37]. 

Among numerous studies of hospitalized and outpatient children worldwide, HMPV has been associated with between 6% and 40% of respiratory illnesses [30]. A study by Edwards et al. in the United States estimated that HMPV was responsible for annual hospitalization rates of approximately 1 per 1000 children under the age of five [33]. In a study by Davis et al. in the United States, hospitalizations were highest for children under the age of 2 years (approximately 2 per 1000 person years). More than 50% of all hospitalized children had an underlying complex chronic condition such as a pulmonary disorder [38,39]; 18% required intensive care and 6% required mechanical ventilation. In a recent study in Norway described by Moe et al. [29,40], up to 10% of children hospitalized with LRT disease were diagnosed with HMPV. In this study, the most severe symptoms were observed among children between the ages of 1 and 2 years, indicating that maternal antibodies provided a degree of protection to infants. Risk factors for serious disease included a history of premature birth or chronic airway disease. A history of premature birth was similarly identified as a risk factor for severe HMPV disease by Pancham et al. in their study of pre-school children [41,42].

Re-infections with HMPV are common, albeit usually mild. The virus threat increases when the human immune system is weakened, as in the context of hematopoietic stem cell transplantation. Aged individuals comprise another vulnerable population due to their waning immune function [36,43,44,45].

## 4. Treatment and Drug Discovery Efforts 

There are no standard treatments for HMPV except supportive care. Ribavirin, a nucleoside analogue, has exhibited anti-HMPV activity in laboratory studies, but its use in clinical studies has yielded conflicting results [29,46,47]. The efficacy, route of administration, and dosing of ribavirin for treatments of respiratory infections have been topics of continuous debate [48]. Other therapies are being developed to block HMPV fusion. For example, the peptide HRA2 (derived from a heptad repeat domain of the HMPV F protein) was shown to significantly reduce viral loads in animal studies [49]. Additionally, small interfering RNAs have been used to block HMPV infections [50,51]. Intravenous immune globulin (IVIG) or HMPV-specific monoclonal antibodies could potentially be used to neutralize HMPV [52], but their benefits as treatment options for HMPV disease have not been proven. For RSV, virus-specific antibodies have been successful as prophylaxes (particularly for vulnerable children including premature infants and infants with heart/lung disease), but not as treatment [53,54,55]. To increase the number of treatment options, methods are now being developed to expedite the screening of anti-HMPV drug candidates [56]. Ongoing studies of HMPV protein structure/function will also assist future drug and vaccine development [57,58].

## 5. Vaccine Development 

Vaccination is the most effective method for control of infectious diseases. If the immune system is primed before a virus exposure, it can eliminate virus in the URT and avoid trafficking of virus to the lung where the greatest damage is caused. 

Several HMPV vaccines have been produced and tested in pre-clinical studies [29,30]. These include recombinant vaccines such as vaccines based on alpha virus or bovine PIV3 [59,60,61]. Killed virus has been tested, although there is a concern that killed virus may exacerbate disease, as was the case in the 1960s when a formalin-inactivated RSV vaccine was clinically tested [62]. Protein, peptide, and virus like particle (VLP) vaccines have also been tried. Live-attenuated HMPV vaccines have been produced, including cold-adapted variants and viruses with mutations in the F or polymerase proteins [30]. 

The HMPV surface glycoproteins G and F have both been tested as vaccine antigens. When F and G genes were individually expressed in recombinant human parainfluenza virus-type 1 (hPIV-1) constructs and tested in hamsters, the F construct exhibited better immunogenicity [63]. In a separate study, when a soluble G protein was expressed (ectodomain without transmembrane domain or cytoplasmic tail), it did not elicit neutralizing antibodies or protection from challenge in cotton rats [64]. In contrast, HMPV F vaccines have been produced using a variety of different vector systems and have repeatedly generated protective immune responses in preclinical animal models [59,60,61].

Sendai virus (SeV) was recently tested as a candidate vector for an HMPV F vaccine [65]. SeV is a mouse parainfluenza virus, closely related to hPIV-1. The recombinant was produced by inserting the HMPV F gene into the SeV backbone between F and HN genes. When the vaccine was delivered intranasally to small research animals, it induced a robust immune response that was protective against an HMPV challenge.

HMPV vaccines have rarely progressed to clinical studies, although a live-attenuated vaccine was once tested in adults [66]. For any new vaccine program, there are numerous steps required to advance from vaccine concept to clinical application. Steps typically include, but are not limited to: (i) vaccine design, (ii) pre-clinical vaccine testing for immunogenicity and efficacy in small and/or large animal models, (iii) preparation of pilot vaccine lots, (iv) stability testing, (v) toxicology testing, (vi) application to institutional and government regulatory boards for approval of vaccines produced with Good Manufacturing Practices (GMP) and associated clinical protocols, (vii) clinical testing of vaccines (phases I, II, III) with regulatory oversight, (viii) license application and approval, (ix) vaccine distribution to the target population using appropriate transport and storage conditions, (x) public acceptance and vaccine use, and (xi) continued vaccine monitoring for safety and efficacy. Lagging development of HMPV vaccines may be due, at least in part, to prioritization of other respiratory virus vaccine programs (e.g., influenza virus, RSV, coronavirus). 

## 6. Raising Awareness

The threat of HMPV infection and disease continues unabated, perhaps because it is not fully recognized. When a respiratory viral infection occurs, it is often assumed to be due to an influenza virus infection and termed ‘flu’. Diagnostics may be available to score influenza virus and possibly RSV, but not HMPV. New multiplex assays that include HMPV are cost prohibitive in many hospitals. Therefore, diseases caused by HMPV will go undiagnosed and unreported. Here, we recommend that efforts be increased in the HMPV field to:

- Advance research efforts to reveal mechanisms by which virus induces a host immune response.
- Improve surveillance to measure the burden of HMPV disease.
- Improve HMPV awareness among clinicians and patients.
- Develop a low-cost diagnostic assay for HMPV.
- Develop new drugs and treatment options for HMPV.
- Advance HMPV vaccines through clinical trials and licensure.

The dedication of new funds and efforts to the conduct of vaccine clinical trials might provide the greatest benefit to the HMPV field by escorting vaccine candidates beyond the stage of pre-clinical testing. A licensed, efficacious vaccine against HMPV could then prevent a substantial number of hospitalizations among children and adults worldwide.

## Abbreviations

Human metapneumovirus, HMPV; respiratory syncytial virus, RSV; parainfluenza virus type 3, PIV-3; Sendai virus, SeV; cytopathic effects, CPE; fusion protein, F; attachment glycoprotein, G; good manufacturing practices, GMP.
